# Neonatal congenital myotonic dystrophy with DMPK gene expansion: clinical features and short-term outcomes

**DOI:** 10.3389/fped.2025.1648611

**Published:** 2026-01-08

**Authors:** Qian Zhao, Shupeng Wang, Yang Wang, Shenggang Ding

**Affiliations:** Department of Pediatrics, The First Affiliated Hospital of Anhui Medical University, Hefei, China

**Keywords:** congenital myotonic dystrophy, DMPK gene, neonatal hypotonia, polyhydramnios, respiratory failure

## Abstract

**Objective:**

To investigate the clinical manifestations, diagnosis and treatment, and DMPK gene mutations in neonates with congenital myotonic dystrophy (CDM).

**Methods:**

A retrospective analysis was conducted on the clinical data of four neonates diagnosed with CDM and admitted to the Department of Neonatology at the First Affiliated Hospital of Anhui Medical University between January 2023 and December 2024.

**Results:**

Among the four cases, three were preterm and one was full-term. Polyhydramnios was noted in the pregnancies of all three preterm infants, and all mothers reported reduced fetal movement. Three preterm infants experienced birth asphyxia. All neonates presented with hypotonia to varying degrees—floppy limbs in preterm infants and marked hypotonia in the full-term infant. All four developed neonatal respiratory failure. Three preterm infants died during the neonatal period, whereas the full-term infant survived following successful weaning and oral feeding. Genetic testing revealed abnormal expansion of (CTG)n trinucleotide repeats in the DMPK gene in all cases, inherited maternally.

**Conclusion:**

CDM should be considered in neonates presenting with unexplained birth asphyxia, hypotonia, and feeding or respiratory difficulties, especially when accompanied by maternal polyhydramnios and reduced fetal movement. Genetic testing enables early diagnosis and intervention.

## 1 Introduction

Congenital myotonic dystrophy (CDM) represents the most severe subtype of myotonic dystrophy type 1 (DM1), with onset during the fetal or neonatal period. The estimated incidence of CDM is approximately 1 in 47,619 live births in Western populations, while epidemiological data in China remain lacking (1).

Clinically, CDM is characterized by a broad spectrum of manifestations. Prenatal signs may include polyhydramnios, reduced fetal movement, and preterm birth. In the neonatal period, affected infants often present with hypotonia, decreased reflexes, generalized muscle weakness, limb contractures, clubfoot, visual impairment, respiratory distress, difficulties with feeding and sucking, cardiac conduction abnormalities, valvular defects, and, in severe cases, sudden death (2). The neonatal mortality rate may reach as high as 40%. Critically ill newborns frequently require immediate resuscitation at birth and prolonged mechanical ventilation due to profound respiratory muscle weakness (3).

A definitive diagnosis of CDM relies on genetic analysis of the DMPK gene, which reveals pathological expansion of (CTG)n trinucleotide repeats (4).

This study represents one of the earliest retrospective clinical analyses of neonatal congenital myotonic dystrophy in China. It focuses on comparing prenatal indicators, birth asphyxia, and short-term outcomes between preterm and full-term infants, and explores the association between polyhydramnios, reduced fetal movement, and neonatal prognosis. Unlike previous reports, which were mostly single cases or narrative reviews, our study provides systematically collected, consecutive cases with detailed clinical and genetic data, offering new evidence for early diagnosis, prenatal counseling, and multidisciplinary management of this rare disorder.

## 2 Materials and methods

### 2.1 Study subjects

This retrospective clinical study included four neonates diagnosed with congenital myotonic dystrophy (CDM) who were admitted to the neonatal intensive care unit (NICU) of the First Affiliated Hospital of Anhui Medical University between January 2023 and December 2024. All cases were genetically confirmed by DMPK gene testing. Since this study included only four neonates with genetically confirmed CDM, it was designed as a retrospective clinical case series using descriptive analysis rather than inferential statistics, which is appropriate for the small sample size and exploratory nature of the research.

Inclusion criteria were: (1) complete perinatal and genetic data available; (2) diagnosis confirmed by abnormal CTG repeat expansion in the DMPK gene; and (3) availability of detailed maternal and neonatal records. The study protocol was reviewed and approved by the institutional Ethics Committee (Approval No. Ethics-PJ 2023-14-25), and written informed consent for participation and publication was obtained from the parents of all participants.

### 2.2 Data collection

Relevant clinical information was retrospectively collected from the hospital's electronic medical records. Data collection included: maternal history (age, gravidity, parity, amniotic fluid volume, fetal movements, medications during pregnancy); neonatal demographics (sex, gestational age, birth weight, mode of delivery, Apgar scores at 1, 5, and 10 min); perinatal manifestations (muscle tone, limb posture, respiratory effort, feeding ability); laboratory and imaging findings (cardiac enzymes, coagulation profile, EEG, ECG, echocardiogram, cranial ultrasound or MRI); and genetic testing results (DMPK CTG repeat number in the proband and the mother). Treatment measures, respiratory support, and short-term outcomes were also recorded.

### 2.3 Diagnostic criteria

The diagnosis of congenital myotonic dystrophy was based on the combination of clinical manifestations (severe neonatal hypotonia, respiratory insufficiency, feeding difficulties, and typical facial features) and genetic confirmation of abnormal CTG trinucleotide repeat expansion in the DMPK gene. For perinatal asphyxia, classification was based on Apgar scores: severe asphyxia (Apgar score ≤3 at 1 min); mild asphyxia (Apgar score 4–7 at 1 min); and no asphyxia (Apgar score ≥8). This grading allowed comparison of neonatal outcomes in relation to the severity of asphyxia.

### 2.4 Genetic testing

Genetic testing for the DMPK gene was performed in all neonates and their mothers. Triplet-primed polymerase chain reaction (TP-PCR) followed by capillary electrophoresis was used to detect CTG repeat expansions. Results were reported in the format of “X/>Y”, indicating the number of repeats in the normal and expanded alleles, respectively (for example, “10/>150” means 10 repeats on the normal allele and >150 on the expanded allele). In clinical interpretation, <35 repeats were considered normal, 35–49 as a premutation, and ≥50 as pathogenic.

## 3 Results

### 3.1 General information

A total of four neonates with genetically confirmed CDM were included in the study. Cases 1 and 2 were extremely preterm infants with gestational ages of 31 + 2 and 28 + 6 weeks, respectively, and both had extremely low birth weights. Case 3 was a late preterm infant at 36 + 1 weeks of gestation, while Case 4 was born at full term.

All three preterm infants were born to mothers with a history of polyhydramnios; two were delivered via cesarean section, and one via spontaneous vaginal delivery. Among them, two mothers had adverse obstetric histories. The full-term infant's mother had normal amniotic fluid volume and underwent vaginal delivery assisted by forceps, with no adverse obstetric history. Decreased fetal movement was reported during pregnancy in all four cases (See Table 1 for a summary of maternal and neonatal characteristics of each case).

**Table 1:** Clinical data summary of 4 neonates with congenital myotonic dystrophy (CDM).

Variable	Case 1	Case 2	Case 3	Case 4
Maternal age (years)	25	32	27	33
Obstetric history	G1, preterm birth, neonatal death due to asphyxia and respiratory failure	G1, embryonic arrest	G1, induced abortion	None
Amniotic fluid	∼1,000 ml	Excessive, unknown volume	∼3,000 ml	Normal
Fetal movement	Decreased	Decreased	Decreased	Decreased
Medication during pregnancy	Levothyroxine	None	Polyene phosphatidylcholine	Levothyroxine/Iron supplements
Gestational age (weeks)	31 + 2	28 + 6	36 + 1	41 + 5
Birth weight (g)	1,460	1,200	2,490	3,500
Sex	Female	Female	Male	Female
Delivery mode	Cesarean	Vaginal	Cesarean	Vaginal (forceps-assisted)
Apgar score 1 min	3	5	3	8
Apgar score 5 min	7	6	3	9
Apgar score 10 min	N/A	8	3	N/A
Main manifestations	Poor response, flaccid limbs, respiratory failure, difficult weaning	Poor response, flaccid limbs, respiratory failure, difficult weaning	Poor response, flaccid limbs, respiratory failure, difficult weaning, cryptorchidism	Low muscle tone, respiratory failure, feeding difficulties, tent-shaped upper lip, frog-leg posture
Other complications	Electrolyte imbalance, ventilator-associated pneumonia	Coagulation dysfunction, metabolic acidosis	Coagulation dysfunction, ventilator-associated pneumonia	Coagulation dysfunction, vitamin D deficiency
CK (U/L)	261	338	230	444
CK-MB (U/L)	335	481	259	63
CSF	Normal	Not performed	Not performed	Not performed
Echocardiography	ASD, PDA	Not performed	ASD, PDA	ASD
Cranial imaging	Normal ultrasound	Not performed	Hyperechoic basal ganglia (suggestive of lenticulostriate vasculopathy)	Subdural hemorrhage in posterior fossa, no parenchymal abnormality
ECG	Sinus rhythm, T wave changes	Normal	Normal	Normal
Other imaging	N/A	N/A	Congenital diaphragmatic elevation	Congenital diaphragmatic elevation
Gene	DMPK	DMPK	DMPK	DMPK
Repeat type	(CTG)n	(CTG)n	(CTG)n	(CTG)n
Proband repeats^a^	10/>150	4/>50	5/>50	11/>150
Maternal repeats^a^	10/>150	4/>50	5/>50	13/>150
Outcome	Died after 9 days of hospitalization	Died after 2 days of hospitalization	Transferred, died days later after withdrawal of care	Discharged after 28 days, survived >5 months with developmental delay

a Normal allele X repeats; expanded allele > Y repeats.

### 3.2 Clinical manifestations and treatment

All three preterm infants experienced birth asphyxia, with two classified as severe. They presented with flaccid limbs, wrist drop, and marked hypotonia, accompanied by respiratory failure and significantly elevated myocardial enzyme levels. Two cases also had coagulation abnormalities, and two presented with congenital cardiac anomalies, including atrial septal defect and patent ductus arteriosus.

The full-term infant did not suffer from birth asphyxia but developed progressive respiratory distress postnatally. Marked hypotonia was noted, along with a tent-shaped upper lip (“inverted V” appearance), persistent frog-leg positioning of the hips (hips abducted with knees flexed), and severe feeding and swallowing difficulties that necessitated respiratory support.

All three preterm infants underwent neonatal resuscitation due to asphyxia, with one case requiring intravenous administration of epinephrine. All four neonates required prolonged mechanical ventilation with difficult weaning. Two of the preterm infants developed ventilator-associated pneumonia and were treated with cefoperazone-sulbactam for infection control.

### 3.3 Genetic testing results

Genetic testing confirmed maternally inherited abnormal CTG trinucleotide repeat expansions in the *DMPK* gene in all four neonates, consistent with congenital myotonic dystrophy type 1 (CDM1).Specifically, Case 1 carried a repeat length of >150 on the expanded allele and 10 repeats on the normal allele, while the mother showed the same expansion pattern. Case 2 exhibited >50 repeats on the expanded allele and 4 repeats on the normal allele; Case 3 had >50 and 5 repeats, respectively;and Case 4 demonstrated >150 repeats on the expanded allele and 11 on the normal allele, with the mother presenting 13/>150 repeats. These data confirm maternal transmission of the pathogenic *DMPK* expansion in all cases.

In general, CTG repeat lengths in congenital DM1 are often very large, frequently exceeding 1,000 repeats; however, in this study, several expanded alleles were reported as “>50”, which may reflect limitations of the detection range inherent to the testing platform rather than smaller true expansions.

### 3.4 Follow-up and prognosis

Cases 1 and 2 died within hours after withdrawal of treatment during hospitalization. Case 3 was transferred to another hospital, where treatment was also discontinued and the patient subsequently died. Case 4 responded well to supportive care, was successfully weaned off mechanical ventilation and oxygen, achieved full oral feeding, and was discharged. At the time of writing, the infant had survived for over five months but exhibited delayed developmental milestones.

## 4 Discussion

Congenital myotonic dystrophy (CDM) represents the most severe end of the DM1 spectrum and is caused by large maternal expansions of CTG repeats in the *DMPK* gene (5). Our 4-case series, although limited in size, provides clinically relevant neonatal data that align with and help contextualize several established observations in the literature.

### 4.1 Principal findings and how they relate to prior reports

The principal observations from our series are (1) frequent prenatal polyhydramnios and reduced fetal movements in affected pregnancies, (2) severe hypotonia with respiratory insufficiency as the dominant early neonatal problem, and (3) markedly worse short-term outcomes in preterm infants and those requiring resuscitation at birth. These findings are concordant with prior reports that identify polyhydramnios as a common prenatal clue to CDM and that emphasize respiratory failure as the major driver of neonatal mortality (6). In particular, prenatal polyhydramnios has been highlighted as a frequent feature in CDM cohorts, supporting the importance of sonographic vigilance when polyhydramnios co-occurs with decreased fetal movements (7).

### 4.2 Prematurity and perinatal asphyxia as prognostic modifiers

Multiple studies have suggested that gestational age at delivery substantially modifies early outcomes in CDM, with preterm infants experiencing far greater respiratory vulnerability (8, 9). Our observation—that the three preterm infants died while the sole term infant survived with multidisciplinary support—echoes these reports and reinforces the clinical message that prematurity is an important negative prognostic factor in CDM. Moreover, our data suggest that the need for resuscitation at birth is associated with particularly poor short-term outcomes; while literature directly quantifying this association is limited, several clinical series and reviews note worse neonatal trajectories when significant perinatal compromise coexists with the muscular and central respiratory impairment of CDM (10). These findings support heightened peripartum preparedness (advanced respiratory support and early rehabilitation) for at-risk pregnancies.

### 4.3 Phenotypic heterogeneity and organ-system findings

Consistent with previous reports, our cases exhibit the characteristic neonatal phenotype—global hypotonia, feeding difficulty, and variable musculoskeletal findings (contractures, possible hip dysplasia, cryptorchidism) (11, 12). Notably, some features (e.g., ventriculomegaly, late-onset ophthalmic changes) may not be apparent in the immediate neonatal period and can emerge later, underscoring the need for longitudinal follow-up. These observations align with natural-history descriptions indicating an evolving multisystem phenotype beyond the neonatal timeline (13).

### 4.4 Genotype–phenotype considerations and technical limits of repeat sizing

The relationship between CTG repeat size and clinical severity is well established in DM1, with congenital cases frequently carrying very large expansions (classically in the thousands), but measurement practices and reporting conventions vary between laboratories (14, 15). Some diagnostic platforms report upper-range values as “>50” or “>150” due to technical sizing limits; such platform thresholds should not be interpreted as implying that an expansion is only modest (16, 17). Therefore, while our genetic results (reported as normal-allele/expanded-allele formats) confirm maternal transmission and support the diagnosis, we explicitly acknowledge that reported “>50” values may reflect assay ceilings rather than true biological upper bounds. When precise sizing is required for genotype–phenotype analyses, Southern blot or long-read approaches (or validated TP-PCR sizing kits) may be necessary.

### 4.5 Clinical implications for prenatal detection and perinatal management

Because many mothers are asymptomatic or undiagnosed carriers prior to pregnancy, CDM may escape prenatal detection unless sonographic and clinical vigilance is applied (18). Our findings support current recommendations that polyhydramnios with decreased fetal movements, especially when combined with other sonographic clues, should prompt genetic counseling and consideration of targeted *DMPK* testing. Early recognition enables multidisciplinary planning (obstetrics, neonatology, genetics) to optimize delivery timing, airway and respiratory preparedness, and immediate neonatal support.

### 4.6 Limitations and future directions

This single-center retrospective series is small and descriptive, so causal inference and statistical generalization are not possible. Follow-up is limited for longer-term neurodevelopmental outcomes. Additionally, non-uniformity in CTG sizing methods limits genotype–phenotype interpretation. To advance the field, prospective multicenter registries with standardized repeat-sizing methods and longitudinal neurodevelopmental assessments are needed to refine risk stratification and to evaluate targeted perinatal interventions.

In summary, our case series corroborates key clinical hallmarks of CDM reported in the literature—notably prenatal polyhydramnios, neonatal hypotonia with respiratory compromise, and marked vulnerability in preterm infants—and highlights the prognostic relevance of gestational maturity and perinatal stability. By explicitly linking our observations with prior studies and clarifying methodological considerations, we aim to improve clinicians' ability to recognize at-risk pregnancies and to prepare appropriate perinatal care pathways.

## 5 Conclusion

This study retrospectively analyzed four neonates with genetically confirmed congenital myotonic dystrophy (CDM) and summarized their clinical, genetic, and prognostic characteristics. Our findings highlight that prematurity and the need for resuscitation at birth are strong negative prognostic indicators in CDM, whereas full-term infants may have a significantly better survival outcome. Polyhydramnios, reduced fetal movement, severe neonatal hypotonia, and respiratory failure should raise early clinical suspicion of CDM, especially in the context of a positive maternal history of myotonia.

Early genetic testing of the DMPK gene plays a crucial role in establishing a definitive diagnosis, enabling timely clinical intervention and accurate family counseling. Prenatal diagnosis remains challenging, but awareness should be heightened among obstetricians, neonatologists, and genetic specialists when these characteristic prenatal or neonatal findings coexist.

This case series confirms the value of integrating perinatal clinical indicators with molecular testing for early recognition of CDM. However, as a single-center retrospective study with a small sample size, the conclusions should be interpreted cautiously. Future multicenter studies with larger cohorts are warranted to further clarify genotype–phenotype correlations and improve prenatal screening strategies for this rare disorder.

## Data Availability

The raw data supporting the conclusions of this article will be made available by the authors, without undue reservation.
